# Immunohistochemical staining reveals differential expression of ACSL3 and ACSL4 in hepatocellular carcinoma and hepatic gastrointestinal metastases

**DOI:** 10.1042/BSR20200219

**Published:** 2020-04-23

**Authors:** Haarith Ndiaye, Jorlin Y. Liu, Andrew Hall, Shane Minogue, Marsha Y. Morgan, Mark G. Waugh

**Affiliations:** 1 UCL Institute for Liver and Digestive Health, Division of Medicine, Royal Free Campus, University College London, London NW3 2PF, U.K.; 2 Sheila Sherlock Liver Centre, Royal Free London NHS Foundation Trust, Hampstead, London NW3 2PF, U.K.; 3 Education Department, Division of Medicine, Royal Free Campus, University College London, London NW3 2PF, U.K.

**Keywords:** fatty acid oxidation, hepatocellular carcinoma, lipid droplets, lipid metabolism

## Abstract

Long-chain fatty acyl CoA synthetases (ACSLs) activate fatty acids by CoA addition thus facilitating their intracellular metabolism. Dysregulated ACSL expression features in several cancers and can affect processes such as ferroptosis, fatty acid β-oxidation, prostaglandin biosynthesis, steroidogenesis and phospholipid acyl chain remodelling. Here we investigate long chain acyl-CoA synthetase 3 (ACSL3) and long chain acyl-CoA synthetase 4 (ACSL4) expression in liver malignancies. The expression and subcellular localisations of the ACSL3 and ACSL4 isoforms in hepatocellular carcinoma (HCC), cholangiocarcinoma (CCA) and hepatic metastases were assessed by immunohistochemical analyses of multiple tumour tissue arrays and by subcellular fractionation of cultured HepG2 cells. The expression of both enzymes was increased in HCC compared with normal liver. Expression of ACSL3 was similar in HCC and hepatic metastases but lower in healthy tissue. Increased ACSL3 expression distinguished HCC from CCA with a sensitivity of 87.2% and a specificity of 75%. ACSL4 expression was significantly greater in HCC than in all other tumours and distinguished HCC from normal liver tissue with a sensitivity of 93.8% and specificity of 93.6%. Combined ACSL3 and ACSL4 staining scores distinguished HCC from hepatic metastases with 80.1% sensitivity and 77.1% specificity. These enzymes had partially overlapping intracellular distributions, ACSL4 localised to the plasma membrane and both isoforms associated with lipid droplets and the endoplasmic reticulum (ER). In conclusion, analysis of ACSL3 and ACSL4 expression can distinguish different classes of hepatic tumours.

## Introduction

Reprogramming of cellular energetics is a hallmark of cancer [1]. Alterations in lipid metabolism are frequently observed during tumour progression and acquired drug resistance [2,3]. Upregulation of fatty acid metabolism can promote cancer survival and proliferation by: (i) providing an alternative to glucose for ATP generation through β-oxidation [4–6] and (ii) driving phospholipid anabolism which is required for increased membrane biosynthesis and oncoprotein-induced cell signalling pathways [7–10].

The liver plays a key role in lipid metabolism. Hepatic lipid dyshomoeostasis is a common prelude to the development of hepatocellular carcinoma (HCC) – one of the world’s most common and lethal cancers [11–13]. This study focuses on the expression and subcellular localisations of two long chain fatty acyl-CoA synthetase family members (ACSLs), ACSL3 and ACSL4, in HCC cells. ACSLs activate fatty acids through ATP-dependent Coenzyme A thioesterification to generate fatty acyl-CoAs that can enter a number of intracellular lipid metabolic pathways [14–17]. ACSL3 and ACSL4 are structurally homologous enzymes; however, they differ in their fatty acid substrate specificities, expression patterns in various tissues and subcellular localisations [8,14,18–20]. ACSL3 preferentially and equally activates palmitic and arachidonic fatty acids, whereas ACSL4 preferentially activates arachidonic acid [21]. ACSL3 localises to the endoplasmic reticulum (ER) and lipid droplets [22–26], but also in some cell types to the *trans*-Golgi network (TGN) [27,28] and insulin-containing secretory granules [29]. ACSL4 is also associated with the ER and lipid droplets [30,31] but endosomal [32], plasma membrane [30], peroxisomal [33] and secretory vesicle [29] localisations have also been reported. Importantly, there is emerging evidence that dysregulated expression of both ACSL3 and ACSL4 is associated with disease and especially with cancer [15,28,34–38]. ACSL3 can promote cancer cell survival through amplified fatty acid β-oxidation [5,37] and increased arachidonic acid-dependent prostaglandin synthesis [39], both of which can drive tumour growth. ACSL4 has also been ascribed functions relevant to oncogenesis; these include ferroptosis, an iron-dependent, non-apoptotic, cell-death pathway [40], metabolic rewiring resulting in drug resistance [35], arachidonic acid-dependent tumorigenesis [41], steroidogenesis [42] and the activation of intracellular, pro-oncogenic signalling pathways [43].

In hepatocytes, ACSL3 expression is controlled by peroxisome proliferator-activated receptor δ (PPARδ) [44], which is activated by arachidonic acid and its metabolites, and required for *de novo* lipogenesis [45,46], lipid droplet formation [26] and very low-density lipoprotein (VLDL) secretion [47]. The role and expression of ACSL3 in HCC has not been studied extensively although a previous analysis of gene expression datasets determined that expression of ACSL3 mRNA was upregulated in this disease [48]. Hepatic ACSL4 expression is also under the control of the PPARδ transcription factor [49,50] and is required for the generation of triglycerides as components of VLDL [51,52]. Previous studies have shown that ACSL4 mRNA levels are increased in approximately 40–80% of HCCs compared with normal liver tissue [53,54]. In addition, inhibitors of ACSL4 expression attenuate the proliferation of a cultured liver cancer cell line [55]. Sun and Xu [56] recently demonstrated that ACSL4 was highly expressed in HCC and that it was a negative prognostic indicator for both disease-free survival and overall survival. Furthermore, a non-biased quantitative proteomic study found that ACSL4 was 1 of 27 proteins that are highly and consistently overexpressed during metabolic reprogramming in HCC [57]. However, ACSL4 expression in non-HCC liver tumours and hepatic metastases has not been previously reported.

Although not the subject of the current work, there is also evidence for dysregulated expression of the ACSL1 and ACSL5 isoforms in HCC. Previous comprehensive studies have demonstrated that ACSL1 expression is increased in HCC patient samples [58,59]. However, in a transgenic murine PTEN knockout, non-alcoholic steatohepatitis (NASH)-induced model for HCC, a quantitative proteomic study found that ACSL1 protein levels were fractionally decreased and ACSL5 levels reciprocally up-regulated [60]. A separate analysis of publicly available large patient datasets reported that mRNA levels for both ACSL1 and ACSL5 mRNA are decreased in HCC [61]. As ACSL1 is robustly expressed both in healthy liver and HCC, and the scenario for ACSL5 is more complex, we decided that these ACSL isoforms would not be useful to pursue as potential IHC markers.

In the present study, we use immunohistochemical analysis of large tissue microarrays to investigate the expression patterns of the homologous ACSL3 and ACSL4 isoforms in a variety of hepatic malignancies with a view to developing a practical tool for the differential diagnosis of HCC.

## Materials and methods

### Materials

Anti-ACSL3 rabbit polyclonal IgG antiserum (catalogue# PA5-42883) was purchased from Thermo Fisher Scientific, U.K.; its specificity has been validated in short hairpin RNAi knockdown experiments [37]. Anti-ACSL4 rabbit polyclonal IgG antiserum (catalogue# 22401-1-AP) was obtained from Proteintech Europe (Manchester, U.K.), its antigen specificity has been validated by both recombinant overexpression and siRNAi studies [36], and it has been used previously for detecting ACSL4 overexpression in HCC [57]. Liver tissue microarrays (#LV2091) were purchased from US Biomax (Rockville, U.S.A.).

### Immunohistochemical staining of tumour microarrays to detect ACSL3 and ACSL4 expression

ACSL3 and ACSL4 expression was investigated using two identical liver tissue microarrays each comprising 208 unstained, formalin-fixed, paraffin-embedded, tissue sections. There was sufficient tissue to allow stain characterisation in 192 of the 208 array samples (Table 1). The remaining 16 samples, comprising 11 HCCs, 4 cholangiocarcinomas (CCAs) and 1 metastasis, could not be processed because there was either insufficient tissue, folding of tissue or an absence of tumour tissue within a cirrhotic/necrotic sample. The microarrays were individually stained with isoform-specific anti-ACSL3 or anti-ACSL4 antisera as described previously [28], and visualised with 3,3-diaminobenzidine (DAB) (a brown stain). The slides were counterstained with Mayer’s Haematoxylin to identify cell nuclei.

**Table 1:** Details of liver tissue microarray samples included in the analysis

**Histopathological diagnosis**	**Number**	**Mean ± D age (year)**	**Sex (M:F)**
**Malignant tissues**			
**HCC**	**141**	**50 ± 8.2**	**119:22 (84%:16%)**
Stage I	8	50 ± 8.0	6:2 (75%:25%)
Stage II	62	48 ± 11.1	52:10 (84%:16%)
Stage III	71	50 ± 13.1	61:10 (86%:14%)
**CCA**	**8**	**39.5 ± 14.1**	**3:5 (38%:63%)**
**Metastatic adenocarcinoma**	**27**	**57 ± 10.4**	**15:12 (56%:44%)**
**Control Tissue**			
**Normal (*n*=8) and normal tumour adjacent (*n*=8)**	**16**	**40 ± 14.1**	**13:3 (81%:19%)**
**Overall total**	**192**	**49 ± 12.3**	**147:45 (77%:23%)**

### Imaging of tumour microarrays

Whole slide imaging magnification was performed using a Hamamatsu NanoZoomer S210 Digital slide scanner (C13239-01) (Hamamatsu Photonics, K.K., Japan). Images were inspected using NanoZoomer Digital Pathology (NDP) viewer software (NDP view.2) (U12388-01) (Hamamatsu Photonics, K.K., Japan).

### Digital image analysis and quantification of staining

Digital images were exported in TIFF format into ImageJ Software (https://imagej.net/ImageJ). Regions of interest (ROIs) were manually selected for each of the 192 samples using the drawing tool to ensure that staining was only quantified from areas of tumour tissue and that areas of fibrotic tissue, necrosis and empty spaces (blood vessel lumen, bile duct lumen) were excluded from staining quantification analysis. The manual selections were subsequently saved as image overlays in ImageJ and were applied to processed images to evaluate the immunohistochemical staining within each ROI.

The images then underwent colour deconvolution using the IHC Toolbox Plugin on ImageJ software [62]. This isolated the Haematoxylin–DAB (H DAB) staining using an intrinsic algorithm, which features defined red, green and blue colour vectors for the particular combination of stain used. The deconvoluted images were subsequently processed to obtain two separate staining metrics viz:

#### Optical density

Deconvoluted images were converted into 8-bit greyscale images. A calibrated optical density (OD) step tablet was used to calibrate the intensity of the grey colour to OD values provided by the ImageJ website [48]. ROI overlays were then applied to the images and the mean intensity of staining was measured within selected ROIs as an OD value.

#### Percentage area positively stained

Staining intensity thresholds for ‘positive’ staining were determined by eye and input into ImageJ. Values of a minimum of 67 and a maximum of 206 were set for analysis of all images. The deconvoluted images were then matched to the thresholds resulting in a binary division of pixels. Pixels which met the threshold were classified as ‘positively stained’, while those that did not were classified as ‘background’. ROI overlays were then applied to the processed images and the proportions of pixels, within selected ROIs, which were ‘positively stained’, were calculated.

The final staining value which was used in all subsequent statistical analysis was the product of these two metrics: OD × percentage area positively stained (%Pos) [80].

### Subcellular fractionation of HepG2 cells on equilibrium sucrose density gradients

HepG2 cells, a HCC cell line [63], was purchased from ATCC (Manassas, VA). Cells were cultured in 15 cm diameter tissue culture dishes in DMEM supplemented with 10% foetal calf serum, penicillin and streptomycin, in a 10 % CO_2_ incubator at 37 °C.

Once confluent cell monolayers had formed, dishes were placed on ice and the medium aspirated. Two dishes per experiment were washed with ice-cold PBS pH 7.4 and scraped into 1 ml of ice-cold cell homogenisation buffer (10 mM Tris/HCl pH 7.4, 0.25 M sucrose and Complete™ EDTA-free protease inhibitors).

The cell suspension was then disrupted using a loose-fitting, hand-held, Dounce homogeniser. The suspension was then centrifuged at 1000 ×*** g*** for 3 min to pellet out nuclei and unbroken cells. The resultant post-nuclear supernatant was then decanted.

The HepG2 post-nuclear supernatant was subsequently separated by ultracentrifugation in an SW41 Beckman swing-out rotor centrifuge at 15000 × *g* at 4 °C in a 15 – 150 % weight/volume sucrose density gradient according to a recently described method [28] designed to isolate different organelles according to their equilibrium buoyant densities. Trial experiments using post-nuclear supernatants prepared from cultured HepG2 revealed that a well-separated, buoyant, lipid droplet fraction was clearly visible after ultracentrifugation at the top of the sucrose density gradient. Following ultracentrifugation, starting at the top of the sucrose gradient; 13 × 1 ml subcellular fractions were harvested.

### Western blot analysis of HepG2 subcellular fractions

Aliquots from each of 13 HepG2 subcellular fractions were combined with an equal volume of 2 X SDS/PAGE sample buffer with DTT reducing agent and then heated for 10 min at 80 °C. 25 μl of samples from each of the 13 fractions were separated by SDS polyacrylamide gel electrophoresis on 12% pre-cast Criterion gels (Bio-Rad Laboratories Ltd, Watford, U.K.). The separated proteins were transferred to PVDF membranes using the iBlot system (Thermo Fisher Scientific, Life Technologies Ltd, Paisley, U.K.). The PVDF membranes were then blocked to minimise non-specific binding using Tris 5 mM, NaCl 137 mM, 0.1 % Tween-29, pH 7.4 buffer (TBST) containing 5 % (w/v) skimmed milk powder for 1–2 h at room temperature. Primary antibodies (Table 2) were added in the dilutions listed and incubated overnight with rotation at 4°C. The following day, the membranes were washed in TBST, refreshing the buffer five times within 30 min. Secondary HRP–conjugated antibodies were added to the membranes at a dilution of 1:10000 to 5% (w/v) skimmed milk/TBST and incubated for 1 h at room temperature. The membranes were then washed five times within 30 min using TBST. Bound antibodies were visualised using the Clarity enhanced chemiluminescence reagents (Bio-Rad Laboratories Ltd, Watford, U.K.) and the FluorChem M gel imaging system (ProteinSimple, Oxford, U.K.), which was also used to quantify signal intensity from Western blots. Each Western blot experiment was repeated two to three times on biological replicate samples.

**Table 2:** Antibodies used in the Western blotting experiments, along with supplier details, the intracellular localisations of their target antigens and the dilutions used in the present study

Antibody	Supplier (catlaogue number)	Antibody dilution
Anti-ACSL4	GeneTex (#GTX100260) (Wembley, U.K.)	1:2000
Anti-ACSL3	Invitrogen (#PA5-42883) (Paisley, U.K.)	1:2000
Anti-calnexin	Invitrogen (#PA5-34754) (Paisley, U.K.)	1:5000
Anti-PNPLA3	Santa Cruz Ltd (#sc-390252) (Wembley, U.K.)	1:500
Anti-flotillin-1	Cell Signaling Technology, Europe B. V. (#18634) (Leiden, The Netherlands)	1:1000
Anti-β-actin	Cell Signaling Technology, Europe B. V. (#4967) (Leiden, The Netherlands)	1:2000
Anti-GS28	BD Biosciences (#611184) (Oxford, U.K.)	1:1000
Anti-syntaxin 6	Cell Signaling Technology, Europe B. V. (#2417) (Leiden, The Netherlands)	1:1000
Anti-EEA1	BD Biosciences (#610457) (Oxford, U.K.)	1:1000
Anti-VDAC	Cell Signaling Technology, Europe B. V. (#4866) (Leiden, The Netherlands)	1:1000
HRP-linked secondary antibodies	Cell Signaling Technology, Europe B. V. (Leiden, The Netherlands)	1:10000

### Data processing and statistical analyses

Sample size and power calculations were performed using the online ClinCalc resource (https://clincalc.com/Stats/SampleSize.aspx) based on 80% power while tolerating a false positive detection rate of 5% and a likely ratio of 10:1 HCC:non-HCC samples. Calculations were based on previously published results, which predicted an ACSL4 overexpression frequency of 80% for HCC samples but not more than 20% for control samples [57]. Using these parameters, a minimum of 43 HCC samples and 4 non-HCC samples would be required for an adequately powered study.

Statistical analyses were performed using *R* for Statistical Computing (version 3.5.2) [64]. Box-whisker plots with dot plots overlaid were created for staining values using ‘boxplot’ and ‘stripchart’ functions. ACSL3 and ACSL4 staining for HCC, metastases, CCA and normal liver tissue were compared using Kruskall–Wallis H tests (one-way ANOVA). Staining levels between groups of tissues were compared using pairwise Wilcoxon rank-sum tests. *P*-values were adjusted for multiple comparisons using the Bonferroni and Hochberg method [65] which limits the false discovery rate. Receiver operating characteristic (ROC) curves were generated to demonstrate performance of ACSL3 and ACSL4 as biomarkers for HCC using pROC package (https://cran.r-project.org/web/packages/pROC/). Optimal cut-off points for staining, sensitivity and specificity values were calculated using the Youden index (optimal threshold is the point on the ROC curve furthest from the diagonal reference line) [66,67].

The combined ACSL3 and ACSL4 biomarker was simulated by performing logistic regression of ACSL3 and ACSL4 against HCC versus control or HCC versus metastases or CCA using the Statistical Package for the Social Science software version 22.0 (IBM, Armonk, NY, U.S.A.) and predicted values obtained, which were then used to produce a ROC curve.

## Results

### Expression of ACSL3 and ACSL4 in a hepatic tissue microarray

**Figure 1:**
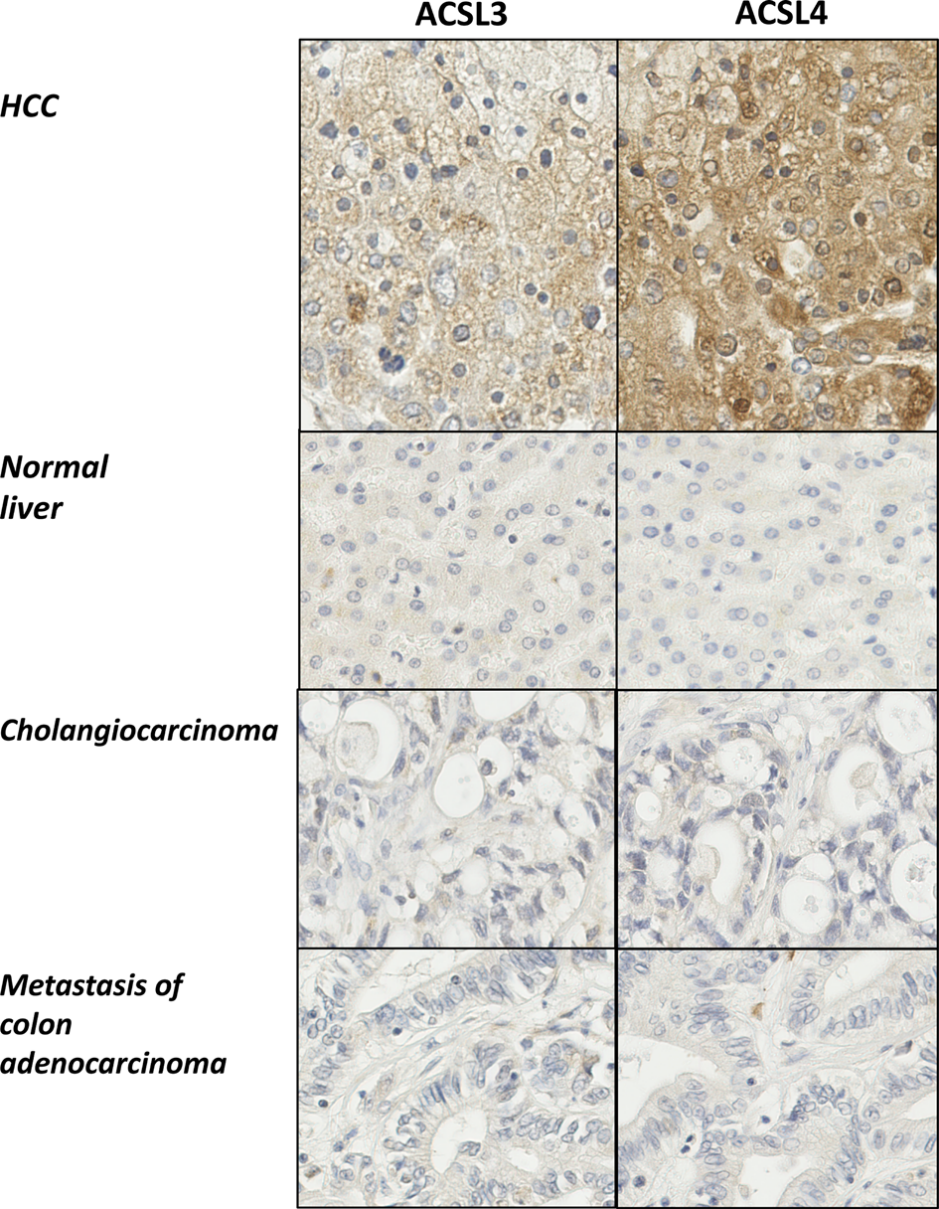
Immunohistochemistry reveals increased expression of both ACSL3 and ACSL4 in HCC Multiple liver tissues arrays were probed with antibodies specific for either ACSL3 or ACSL4. Representative examples (×20 maginfication) are shown for either ACSL3 or ACSL4 immunohistochemical staining of matched samples of HCC, normal liver, CCA and liver metastases.

**Figure 2:**
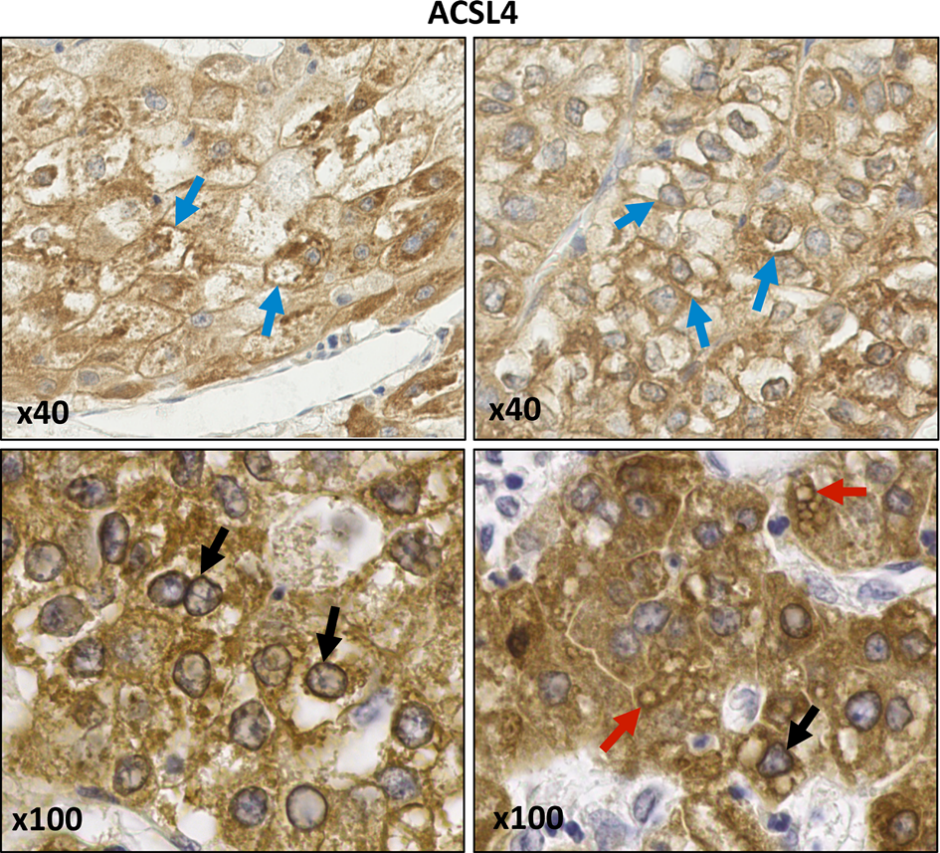
Anti-ACSL4 IHC staining of HCC samples ACSL4 staining is visible at plasma membranes (blue arrows), perinuclear membranes (black arrows) and on lipid droplets (red arrows). Images were obtained at either ×40 or ×100 magnification.

**Figure 3:**
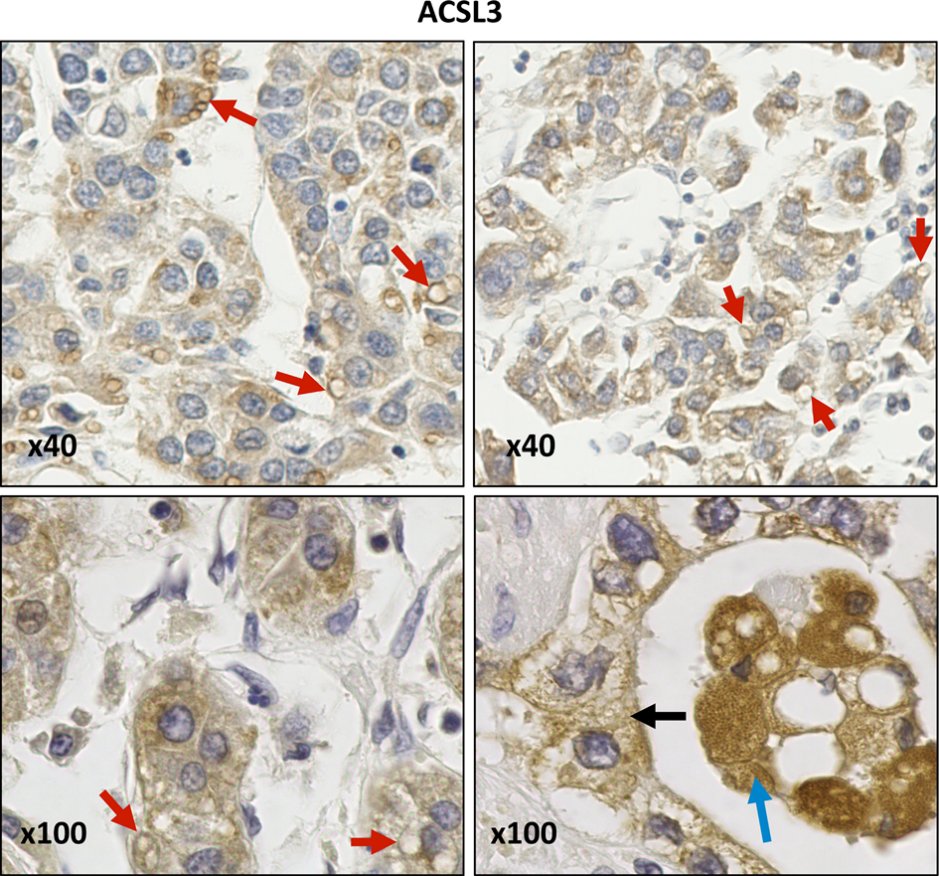
Anti-ACSL3 IHC staining of HCC samples ACSL3 staining is present on lipid droplets (red arrows) and cytoplasmic reticular membranes (black arrow). Images were obtained at either ×40 or ×100 magnification.

Immunohistochemical staining for both isoforms exhibited cytoplasmic membrane localisation patterns that tended to be more intense and extensive in HCC samples compared with normal liver tissue, CCA or hepatic metastases (Figure 1). In HCC samples, ACSL4 was prominent at the plasma membrane, cytoplasmic granules, on the surface of lipid droplets and on perinuclear membranes (Figure 2). In addition, cytoplasmic reticular staining was evident (Figures 1 and 2). ACSL3 staining was also present on the surface of lipid droplets and cytoplasmic reticular structures but unlike ACSL4, it was not visible at the plasma membrane (Figure 3). Neither ACSL3 nor ACSL4 were detected on the surface of lipid droplets in either normal liver tissue or in non-HCC tumour cells in any of the samples examined.

### Quantitative analysis of ACSL3 and ACSL4 immunohistochemical staining

**Figure 4:**
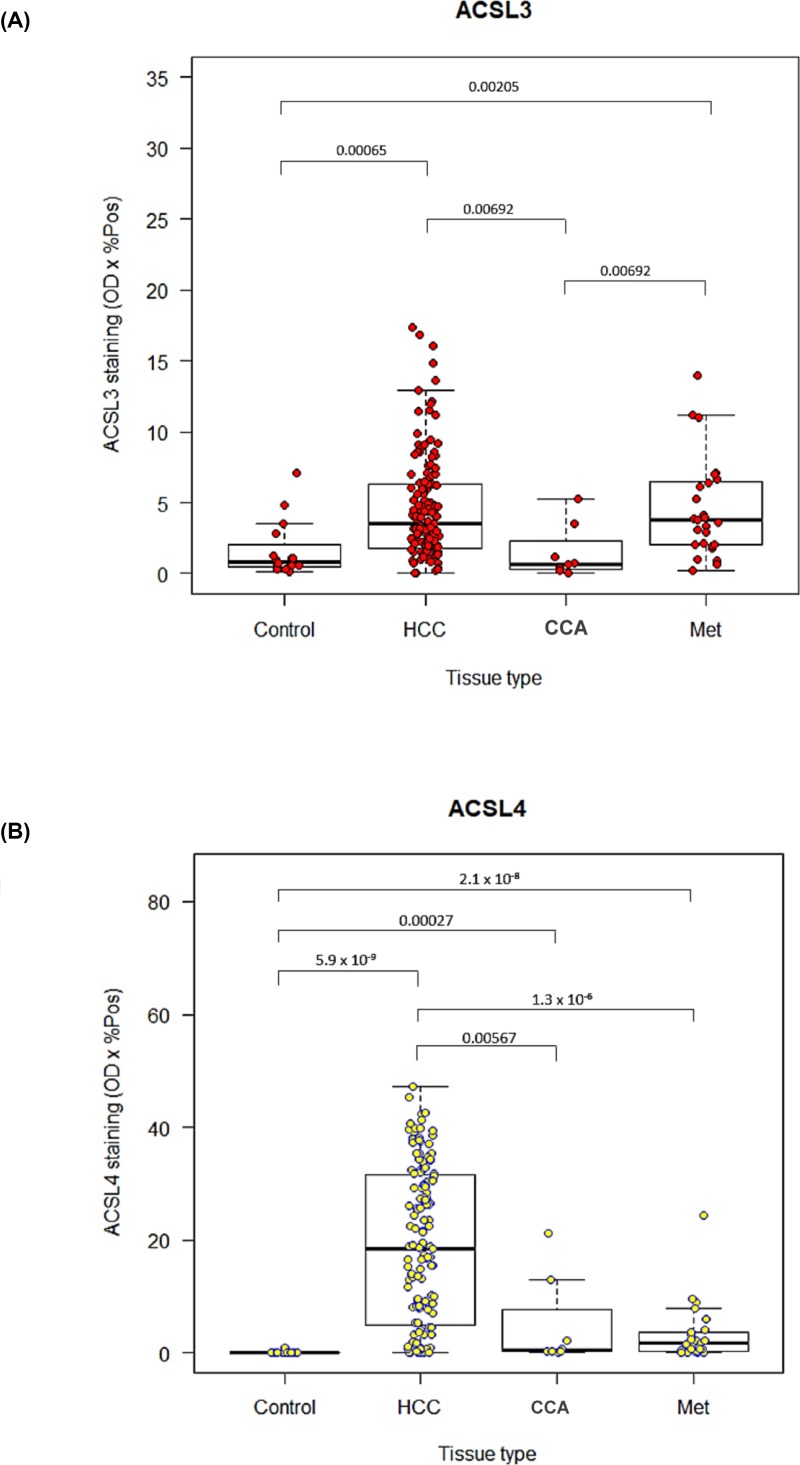
Expression of ACSL3 and ACSL4 enzymes in hepatic malignancies Computer-aided, quantitative image analysis of (**A**) ACSL3 and (**B**) ACSL4 immunohistochemical staining of a liver tissue microarray for healthy controls, HCC, CCA and hepatic metastases (Met). Box-whisker plots with dot plots overlaid showing the median, interquartile range, minimum and maximum values, ACSL3 and ACSL4 staining values. Pairwise Wilcoxon rank sum tests were performed, significant *P*-values for which are shown on the graphs.

A box-whisker plot presenting ACSL3 immunohistochemical staining values for control tissues, HCC, CCA and metastases is presented in Figure 4A. A Kruskal–Wallis H test (one-way ANOVA) showed that there was a statistically significant difference in staining values between tissue types, χ^2^ (3) = 35.40, *P*<0.0005 (mean rank staining was 60.0 for control tissue, 128.0 for HCCs, 134.5 for metastases to the liver and 67.10 for CCA).

To further investigate these differences, pairwise Wilcoxon rank sum tests comparing ACSL3 staining between individual pairs of tissue types were carried out, adjusting for multiple comparisons (Bonferroni and Hochberg method [65]). First, all HCC tissues were compared against controls, CCAs and metastases to the liver. Significant *P*-values are displayed in Figure 4A. ACSL3 staining was significantly higher for HCCs compared with control tissues (*P*=0.00065) and CCAs (*P=*0.00692), but not compared with liver metastases. Metastases also had significantly higher ACSL3 staining than both CCA (*P=*0.00692) and control tissue (*P*=0.00205). The results show that an elevated level of ACSL3 is not a specific feature of HCC. However, tissue samples with higher ACSL3 staining are much more likely to be cancerous than normal.

Box-whisker plots of ACSL4 staining for different tissue types reflect our initial observations of the whole slide imaging in that staining values for HCCs are much higher than for all other tissue types (Figure 4B). Moreover, there is much greater variation in HCC staining values compared with controls, CCAs and metastases, which all seem to be concentrated towards the lower end of staining.

Kruskal–Wallis H test (one-way ANOVA) showed that there was a statistically significant difference in staining values between tissue types, χ^2^ (3) = 63.60, *P*<0.0005 (mean rank staining was 14.63 for control tissue, 114.5 for HCCs, 61.63 for metastases to the liver and 60.25 for CCAs).

Thus, pairwise Wilcoxon-rank sum tests were carried out to test staining differences between tissue types adjusting for multiple comparisons, the significant results of which are shown in Figure 4B. As expected, HCC tissues had significantly higher ACSL4 staining than controls (*P*=5.9 × 10^−9^), CCAs (*P*=0.0057) and metastases to the liver (*P*=1.3 × 10^−6^). In addition, all cancerous tissues manifested significantly higher ACSL4 staining compared with controls.

**Figure 5:**
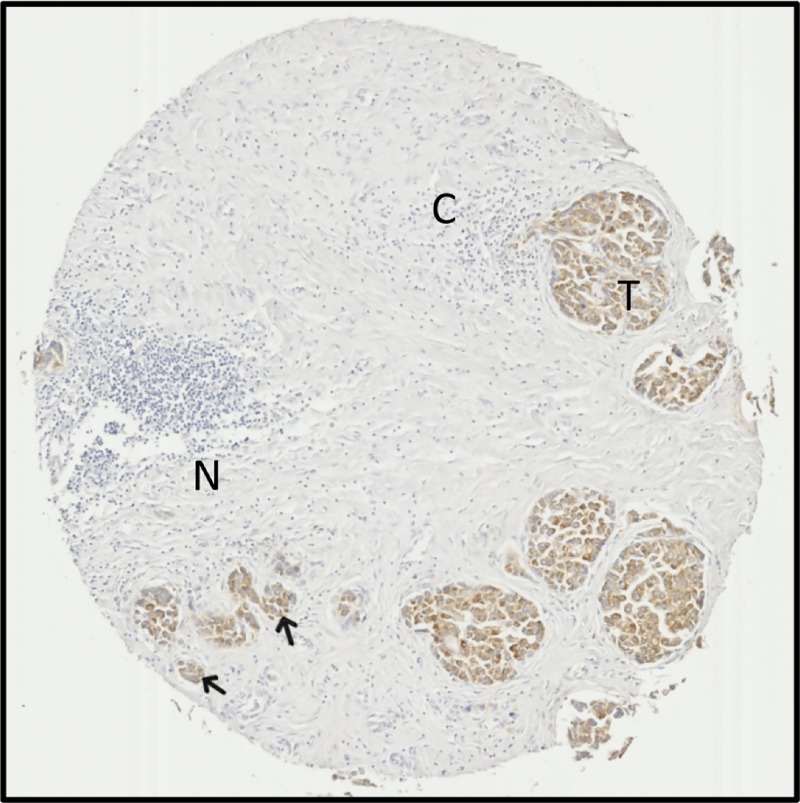
ACSL4 staining highlights tumour tissue amidst extensive cirrhosis and necrosis in samples Tissue microarray sample from a 60-year-old male with stage II HCC. ACSL4 staining is apparent in tumour regions (T) and small tumour tissue foci (arrows), but absent from regions of cirrhotic tissue (C) or necrosis (N). Images are ×5 magnified.

Overall, high ACSL4 staining seems to be highly specific to HCC tissues, demonstrating a potential to differentiate between normal tissue, CCA and metastases within the liver. Furthermore, ACSL4 staining is useful to distinguish small clusters of HCC cells from a background of substantial cirrhosis and necrosis (Figure 5).

### Diagnostic performance of ACSL3 and ACSL4 immunohistochemical staining

The performance of ACSL3 expression for distinguishing HCC from both normal tissue (area under the curve (AUC) 0.796; CI (0.669 – 0.923); sensitivity 85.8 %; specificity 75.0 %) and CCA (AUC 0.803; CI (0.624–0.963); sensitivity 87.2; specificity 75.0 %) was good (Table 3). However, its performance in distinguishing HCC from hepatic metastases lacked specificity (AUC 0.552; CI (0.439–0.665); sensitivity 87.2 %; specificity 28.6 %).

**Table 3:** Performance of the immunohistochemical staining for ACSL3 and ACSL4 expression in liver cancer tissue microarrays for the diagnosis of HCC

ROC curve comparisons	AUC (95% CI)	Optimal threshold	Sensitivity (%)	Specificity (%)
**ACSL3, HCC vs. normal liver**	0.796 (0.669–0.923)	1.29	85.8	75.0
**ACSL3, HCC vs. cholangiocarcimoma**	0.803 (0.624–0.963)	1.18	87.2	75.0
**ACSL3, HCC vs. hepatic metastases**	0.552 (0.439–0.665)	1.18	87.2	28.6
**ACSL4, HCC vs. normal liver**	0.967 (0.939–0.995)	0.12	93.8	93.6
**ACSL4, HCC vs. CCA**	0.796 (0.672–0.923)	2.77	80.1	75.0
**ACSL4, HCC vs. hepatic metastases**	0.801 (0.736–0.867)	13.00	62.4	94.3
**Combined ACSL3 & ACSL4, HCC vs. normal liver**	0.972 (0.945–0.998)	n/a	84.4	100.0
**Combined ACSL3 & ACSL4, HCC vs. CCA**	0.849 (0.735–0.964)	n/a	61.7	100.0
**Combined ACSL3 & ACSL4, HCC vs. hepatic metastases**	0.801 (0.762–0.890)	n/a	80.1	77.1

Abbreviation: n/a, not applicable.

The performance of ACSL4 expression for distinguishing HCC from both normal tissue (AUC 0.967; CI: (0.939–0.995); sensitivity 93.8 %; specificity 93.6 %) was excellent, and it performed well in distinguishing HCC from CCA (AUC 0.796; CI (0.672–0.923); sensitivity 80.1 %; specificity 75.0 %). Immunohistochemical staining of ACSL4 was less effective at distinguishing HCC from hepatic metastases (AUC 0.801; CI (0.736– 0.867); sensitivity 62.4 %; specificity 94.3 % (Table 3)).

Combining ACSL3 and ACSL4 expression did not provide significant additional advantage over the expression of ACSL4 alone in distinguishing HCC from healthy tissue or metastases. However, combining ACSL3 and ACSL4 staining did improve performance for distinguishing HCC from CCA (AUC 0.801; CI (0.762–0.89); sensitivity 80.1 %; specificity 77.1 % (Table 3)).

### Subcellular fractionation of HepG2 cells to investigate the intracellular distributions of ACSL3 and ACSL4

**Figure 6:**
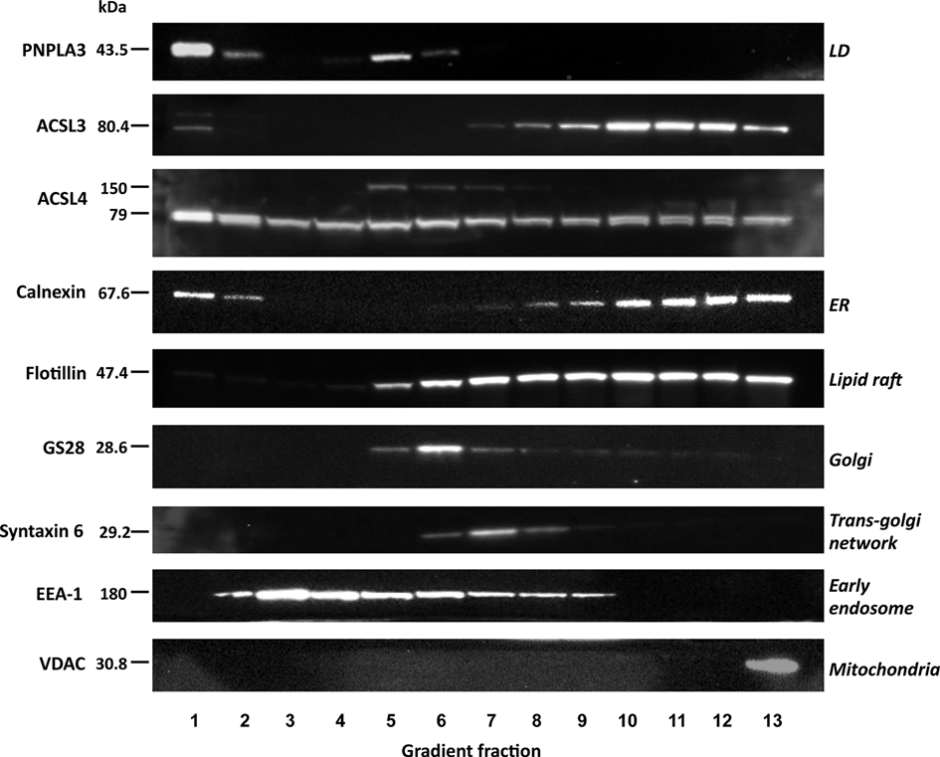
Equilibrium distributions of ACSL3 and ACSL4 in sucrose density gradient fractions prepared from HepG2 cells Subcellular fractions isolated from HepG2 cells were separated by SDS/PAGE and Western blots were carried out to detect the lipid-droplet protein PNPLA3, ACSL3 and ACSL4, the ER marker protein calnexin, plasma membrane and lipid-raft associated flotillin, the Golgi protein GS28, the TGN-endosomal protein syntaxin-6, the early endosome-recruited protein EEA and the mitochondrial protein VDAC. Western blots are representative of experiments repeated three to four times.

A well-separated, buoyant, lipid droplet fraction was clearly visible at the top of equilibrium sucrose density gradients following ultracentrifugation of HepG2 post-nuclear supernatants. Western blotting confirmed that this fraction was highly enriched for the lipid droplet marker protein PNPLA3 (Figure 6). Western blotting of gradient fractions with a panel of organelle marker proteins confirmed that the lipid droplet fraction was well separated from flotillin - a marker for lipid rafts of the plasma membrane and TGN [68,69]; syntaxin-6 a marker for the TGN and early endosomes; EEA1 a marker for early endosomes; GS28 a Golgi marker; VDAC a protein marker for mitochondria and calnexin – a protein principally found at the ER. A small pool of calnexin was also detected in the lipid droplet fraction and this is consistent with previous work [70].

ACSL3 and ACSL4 had distinctive distribution profiles in the density gradients. Semi-quantitative analysis of ACSL3/4 distributions in HCC cell fractions demonstrated that approximately 10% of the total cellular compliment of each isoform was stably associated with the PNPLA3-enriched lipid droplet fraction. Furthermore, the bulk of the cellular ACSL3 closely co-fractionated with the ER marker calnexin, suggesting that this enzyme mainly localises to this major lipid synthesising compartment in HCC cells. The distribution of ACSL4 in the density gradient fractions mirrored the localisation patterns observed in immunohistochemical imaging with a more widespread intracellular distribution than ACSL3. In addition to PNPLA3-enriched lipid droplets and denser calnexin-containing fractions, substantial ACSL4 immunoreactivity was present in intermediate density fractions containing the plasma membrane, TGN and endosomal compartments. Occasionally, in some anti-ACSL4 blots, an additional, non-specific band at approximately 160 kDa was observed in some density gradient fractions but this was not a reproducible finding.

## Discussion

In the present study, the expression of the fatty acid activating enzymes ACSL3 and ACSL4 was found to be significantly upregulated in HCC cells. We observed that ACSL3 expression was increased in HCC and to a lesser extent in hepatic metastases. These findings are consistent with a general augmentation of ACSL3 levels in hepatic malignancies but clearly limit the usefulness of ACLS3 as an independent immunohistochemical marker for identifying HCC, although the differentiation of HCC from CCA was good.

In concordance with previous reports [53,54], we found that ACSL4 expression was significantly increased in HCC tissues compared with normal liver, distinguishing the two with a sensitivity of 93.8% and a specificity of 93.6%. In addition, increased ACSL4 expression distinguished HCC from both CCA and hepatic metastases. It is important to note that altered expression of ACSL3 and ACSL4 is not unique to hepatic malignancies. Up-regulated ACSL4 expression has been previously characterised in extrahepatic cancers such as colon adenocarcinoma [71], lung [5,39], breast [34,35,41,72,73] and prostate cancers [72]. Increased ACSL4 expression is a determinant of drug resistance in metastatic breast cancer cells where altered cellular energetics leads to increased expression of the ATP-binding cassette (ABC) transporter, which mediates the egress of chemotherapeutic molecules [35]. A similar scenario exists for prostate cancer where increased ACSL4 is associated with increased aggressiveness, therapeutic resistance and enhanced anti-apoptotic signalling [43,72]. Interestingly, for both prostate and breast cancers increased ACSL3 is also associated with particular tumour subtypes [37,74–76]. These precedents may indicate that up-regulation of either isoform in liver cancers may contribute to an oncogenic phenotype.

A correlation between amplified ACSL3/4s expression and increased malignancy is not, however, universal. Particularly so since ACSL4 is required for ferroptosis which has a potential tumour-suppressive function. As an example, ACSL4 is significantly down-regulated in gastric cancer compared with cancer-adjacent normal gastric mucosa [36]. Moreover, transient overexpression of recombinant ACSL4 in gastric cancer cell lines significantly inhibited cell growth, proliferation and migration *in vitro*, whereas knockdown of ACSL4 induced reciprocal effects [36]. Taken together, these examples point to tumour subtype-specific functions for these enzymes in oncogenesis. With regard to HCC, previous work has demonstrated that increased ACSL4 may be a determinant of drug resistance [77] and increased tumour growth [53,55]. By comparison, the role of ACSL3 in hepatic malignancies has been less well studied but it is tempting to speculate that it may potentially drive tumorigenesis through increased mitochondrial fatty acid β-oxidation [5]. However, future functional experiments are required in order to delineate the functional consequences of altered ACSL3 and ACSL4 expression in the different classes of liver tumours that were investigated in the present study.

The combined ACSL3 and ACSL4 biomarker simulated in the present study performed well to distinguish between HCC from hepatic metastases with a sensitivity of 80% and specificity of 77%. The samples in the tissue array included gastrointestinal carcinomas that commonly metastasise to the liver such as colon, pancreas and stomach [78]. The combined ACSL3 and ACSL4 staining have a similar sensitivity for distinguishing HCC from hepatic metastases as Arginase-1 [79] and the hepatocyte membrane transporter proteins BSEP and MDR3 [80]. The liver is one of the most common sites for metastatic disease; metastases occurs more frequently in the liver than HCC in many European countries and in the United States [78]. The metastases included in this tissue array were gastrointestinal in origin so the performance of ACSL3 and ACSL4 combined biomarker will need to be assessed in relation to other common metastatic tumours such as those arising from lung, breast and melanoma. It will also be necessary to determine whether this combination marker can distinguish HCC in more challenging clinical scenarios such as mixed hepatocellular-CCA or rarer HCC mimetic tumours such as hepatocellular adenoma or hepatoid adenocarcinoma [81].

The mechanism of ACSL3 and ACSL4 overexpression in HCC remains to be elucidated. Data from the Catalogue of Somatic Mutation in Cancer (COSMIC) v89 [82] indicated that amongst hundreds of HCC samples tested, 0.33% (3/899) had point mutations in the *ACSL4* gene, 0.73% (5/682) had copy number variations and 11.8% (44/373) had upregulated gene expression. Similar results were found for ACSL3. Thus, although gene expression may be partially responsible for the increased levels of these enzymes in HCC, it is likely that their upregulation is predominantly due to factors relating to their transcription, translation and possibly degradation, most likely under the control of established oncoproteins such as KRAS [5] or lipid-activated transcription factors such as PPARδ [44,50]. Further investigations into the mechanism underlying dysregulated oncogenic expression of ACSL3 and ACSL4 expression in HCC may identify new drug therapeutic targets for drug development.

One consistent finding in the present study was the strong immunohistochemical stationing of both ACSL3 and ACSL4 on the surface of intracellular lipid droplets suggesting that in HCC, both enzymes are involved in fatty acid metabolism on these lipid storage organelles [83]. Recent work suggests that nuclear lipid droplets in hepatocytes result from endoplasmic reticulum stress [83]. Furthermore, ACSL3 expression is upregulated in liver cells in response to ER stress [84]. In such instances, ACSL3/4 overexpression and lipid droplet association may represent an adaptive response. However, it is important to note that while there is substantial evidence that increased intracellular lipid storage can define a more aggressive class of cancers (reviewed in [85,86]), the association of both enzymes with lipid droplets in HCC cells does not necessarily imply that this forms part of an oncogenic process.

Interestingly, two splice variants of *ACSL4* referred to as ACSL4-v1 or ACSL4-v2, have been identified. Both splice variants have the same biochemical activities but differ in their subcellular distributions with the shorter ACSL4-v1 being targeted to the plasma membrane and cytosol, whereas the longer ACSL4-v2 is primarily associated with lipid droplets [30]. However, the reagents used in this study did not distinguish between the different splice variants and hence it is not possible to infer that the different pools of ACSL4 observed were due to structural variations in this isoform. On the other hand, ACSL3 has a much simpler subcellular distribution profile and was largely absent from the intermediate density fractions containing the plasma membrane and endosomal compartments. The gradient distribution profile of ACSL3 immunoreactivity closely followed that of the predominately ER-resident protein calnexin. A small pool of ACSL3 was also associated with the PNPLA3-positive lipid droplet fraction and this gradient distribution aligns well with previous reports on the subcellular compartmentalisation of this enzyme [23,31].

In conclusion, our results demonstrate that amplified expression of ACSL3 and ACSL4, and their increased association with lipid droplets is a feature of HCC. These results have a use in distinguishing HCC from other liver tumours an also suggest that upregulated fatty acid metabolism is a potential chemotherapeutic target for the treatment of HCC.

## Abbreviations

ACSL: long-chain fatty acyl CoA synthetase
ACSL3: long chain acyl-CoA synthetase 3
ACSL4: long chain acyl-CoA synthetase 4
CCA: cholangiocarcinoma
DAB: 3,3-diaminobenzidine
ER: endoplasmic reticulum
HCC: hepatocellular carcinoma
MET: metastasis
OD: optical density
PPARδ: peroxisome proliferator-activated receptor δ
ROC: receiver operating characteristic
ROI: region of interest
TGN: *trans*-Golgi network
